# Physician-estimated disease severity in patients with chronic heart or lung disease: a cross-sectional analysis

**DOI:** 10.1186/1477-7525-4-60

**Published:** 2006-09-13

**Authors:** Kurt Kroenke, Kathleen W Wyrwich, William M Tierney, Ajit N Babu, Fredric D Wolinsky

**Affiliations:** 1 Indiana University School of Medicine, Regenstrief Institute, 1059 Wishard Blvd, Indianapolis, IN 46202, USA; 2 School of Public Health, Saint Louis University, 3750 Lindell Blvd. McGannon Hall, Room 230, USA; 3 Division of General Internal Medicine and Geriatrics, Indiana University School of Medicine, 1050 Wishard Blvd, Indianapolis, IN 46202, USA; 4 Amrita Institute of Medical Sciences, Kochi, India; 5 College of Public Health, The University of Iowa, 200 Hawkins Drive, E205-GH, Iowa City, Iowa 52242, USA

## Abstract

**Background:**

We evaluated how well physicians' global estimates of disease severity correspond to more specific physician-rated disease variables as well as patients' self-rated health and other patient variables.

**Methods:**

We analyzed baseline data from 1662 primary care patients with chronic cardiac or pulmonary disease who were enrolled in a longitudinal study of health-related quality of life (HRQoL). Each patient's primary physician rated overall disease severity, estimated the two-year risk of hospitalization and mortality, and reported the use of disease-specific medications, tests, and subspecialty referrals. Patient variables included sociodemographic characteristics, psychosocial factors, self-rated health, and both generic and disease-specific HRQoL.

**Results:**

Physicians rated 40% of their patients "about average", 30% "worse", and 30% "better" than the typical patient seen with the specific target disorder. The physician's global estimate of disease severity was strongly associated (*P *< 0.001) with each of the five more specific elements of physician-rated disease severity, but only marginally associated with patient self-rated health. Multivariable regression identified a set of patient variables that explained 16.4% of the variance in physician-rated disease severity.

**Conclusion:**

Physicians' global ratings may provide disease severity and prognostic information unique from and complementary to patient self-rated health and HRQoL measures. The elements influencing physician-rated disease severity and its predictive validity for clinical outcomes warrant prospective investigation.

## Background

Many patients suffer from one or more chronic diseases the severity of which can influence both present health (symptoms, functional status, and quality of life) as well as future health-related events (morbidity, mortality, health care use). Cardiac and pulmonary disease are among the most common chronic medical disorders and account for substantial disability, mortality, and health care utilization. A variety of methods may be used to gauge disease severity including (but not limited to) objective measures (e.g., biological, physiological, anatomical, functional), expert clinician judgment, and patient-reported health-related quality of life (HRQoL) measures. The latter have proven particularly promising for predicting a variety of outcomes [1-8].

Among these various methods of measuring disease severity, it is well documented that the traditional single-item patient self-rated health question (How would you rate your health – would you say it is excellent, very good, good, fair, or poor?) is consistently a strong independent predictor of future outcomes, including mortality, disability, and health care utilization [9-11]. Simply put, the self-rated health question is a powerful "gestalt" measure of the patient's health status. Fewer studies have been done on the physician's global estimate of their patients' health and disease severity. This is surprising, because one would expect that a physician-rated "gestalt" question would complement the patient self-rated assessment given the physician's clinical training and objectivity, coupled with the physician's ability to integrate multiple items of data from the history, physical examination, and diagnostic tests and procedures. Two previous studies comparing physician and patient global estimates had conflicting results [12,13]. In several other studies the sole emphasis has been the physician's predictive accuracy in special populations, such as the short-term prognosis in seriously ill patients admitted to intensive care units or survival in patients with terminal illness, usually cancer [14-19]. A better understanding of physicians' prognostic estimates in patients with chronic medical disorders is important in that the longitudinal care of such disorders constitutes a substantial part of many physicians' practices.

In order to pursue a single-item physician "gestalt" measure of the patient's disease severity, we gathered baseline data that allowed us to consider both the patient's and the physician's views of disease severity as part of a longitudinal study of HRQoL in a large cohort of patients with chronic cardiac or pulmonary disease. Using these data, in this paper we address three major questions:

1. How well does the primary care physician's global estimate of disease severity correspond to more specific elements of disease severity, namely estimates of the projected two-year risk of hospitalization and mortality and the use of disease-specific medications, tests, and subspecialty referral? That is, are physicians internally consistent with their severity estimates?

Also, hospitalization as well as ordering medications, tests, and referrals are concrete actions frequently taken by clinicians in response to disease severity. Thus, an association between these actions and the physician's global disease severity estimate demonstrates convergent validity.

2. What is the concordance between disease severity as rated by the physician and the patient's own self-rated health?

3. What patient variables correlate with physician-estimated disease severity?

Although our ultimate aim is to determine the predictive validity of physician global "gestalt" estimates of the patient's disease severity, our cross-sectional analyses are an initial step in establishing the strengths and limitations of this approach.

## Methods

### Study sample

This paper uses data from a large longitudinal study of HRQoL among older adults with coronary artery disease and/or congestive heart failure (CAD/CHF), chronic obstructive pulmonary disease (COPD), or asthma. Subjects were recruited from the adult primary care outpatient practices at the Indiana University School of Medicine and the Saint Louis Veterans Affairs Medical Center.

With the use of electronic medical records, patients were identified as being potentially eligible based on age and medical criteria. Medical criteria for the three target disease groups were specified by three expert panels of North American physicians [20-22]. For asthma, patients 18 years or older were eligible while for CAD/CHF and COPD, patients needed to be 50 years or older. The 46 primary care physicians for these patients then reviewed the specific information for each of their patients and indicated whether or not the patients had the target diseases. Attempted enrollment was limited to the 2,493 patients confirmed by their primary care physician to have one of the target disorders and who kept scheduled primary care visits during August 2000 to November 2001. Of these, 1,662 (66.7%) were enrolled and interviewed at baseline.

### Physician-reported disease severity variables

Primary care physicians completed a baseline questionnaire on all but 4 study patients, for a completion rate of 99.8%. This 6-item questionnaire included a global or "gestalt" estimate of disease severity plus 5 questions about the probability of future hospitalization and death, and the use of medications, testing, and specialty referral:

1. Compared to other patients that you see with <target disease>, how serious is this patient's <target disease>? Response options were: 1 = much worse; 2 = somewhat worse; 3 = about average; 4 = somewhat better; 5 = much better.

2. What is the chance (to the nearest 10%) that the patient will be hospitalized for <target disease> in the next 2 years?

3. What is the chance (to the nearest 10%) that the patient will die, directly or indirectly due to target disease>, in the next 2 years?

4. Is this patient on medication(s) for his/her <target disease> (1 = yes, 0 = no)?

5. As far as you know, has the patient had laboratory tests or procedures ordered because of his/her <target disease> (1 = yes, 0 = no)?

6. As far as you know, has this patient seen a specialist for his/her <target disease> (1 = yes, 0 = no)?

### Other variables

Demographic factors include age (coded in years), a binary marker for men (vs. women), and a set of two dummy variables (black, and non-black non-white, vs. white) for race. Socioeconomic characteristics included education, employment history, and subjective income. Education was measured in years of completed schooling (range = 0 to 25). Employment history was measured by a set of two dummy variables reflecting working for pay or being retired (vs. no substantial history of labor force participation). Subjective income was measured by a set of two dummy variables: comfortable income or not enough income (vs. just enough to get by).

Psychosocial factors included social support, stress, religiosity, sense of control, long-term smoking, and patient satisfaction. Social support was measured by a 5-item subset (alpha = .849) of the Medical Outcomes Study social support scale [23], transformed such that zero reflects the least support and 100 reflects the greatest support. Stress was measured with the National Health Survey 2-item personal stress scale (alpha = .681) from the National Opinion Research Center [24], transformed such that zero reflects maximal stress and 100 reflects minimal stress. Religiosity was measured by a two-item scale (alpha = .793) using the summary religiosity and spirituality items from the Fetzer instrument [25], transformed such that zero reflects the least religiosity and 100 reflects the greatest religiosity. Sense of control was assessed with Mirowsky and Ross' 8-item (alpha = .690) measure [26], where -16 reflects positions of maximal fatalism, +16 reflects positions of maximal responsibility, and 0 reflects balance. Long-term smoking was measured by a binary variable for ≥ 20 pack years (vs. less or none). Patient satisfaction was assessed using a 10-item scale (alpha = .950), transformed to range from 0 (low) to 100 (high).

A binary marker for the public hospital clinics in Indianapolis (vs. the Veterans Affairs Medical Center clinics in St. Louis) was included to evaluate differences between the two enrollment sites. A set of two dummy variables (asthma, and COPD, vs. CAD/CHF) was included in modeling to reflect target disease differences.

Generic health-related quality of life (HRQoL) was assessed with the SF-36, which measures eight domains: physical functioning, mental health, social functioning, bodily pain, vitality, general health perceptions, and physical role and emotional role functioning [27]. On each SF-36 scale, zero reflects the worst and 100 the best score. Disease-specific impact on activities was assessed with the chest pain/shortness of breath scale from the modified Chronic Heart Failure Questionnaire [28] for the CAD/CHF patients, shortness of breath scale from the Chronic Respiratory Questionnaire [29] for the COPD patients, and activity limitation scale taken from the Asthma Quality of Life Questionnaire [30] for the asthma patients. Each of these disease-specific instruments includes 5 items that ask patients to select the five most important activities in their daily lives that are impacted by their target disease and estimate to what degree they have been limited during the past four weeks in each of these five activities on a 1 (severely limited) to 7 (not limited at all) scale. Thus, the five-item scale scores for disease-specific impact on range from 5 to 35 (bad to good).

### Statistical analysis

Physician responses to the 6 disease severity questions were described using means for continuous and proportions for categorical variables. Associations among the physician-reported disease severity variables and between physician-reported measures and patient self-rated health were tested using chi-square analyses.

To determine which patient variables were independently associated with the physician's global estimate of disease severity, we conducted stepwise ordinary least squares (OLS) regression analysis in which severity was treated as a continuous variable from 1 to 5, and multinomial multiple logistic regression analysis in which severity was treated as a categorical variable (with five response outcomes). The modeling process sequentially entered the demographic factors (block 1); socioeconomic characteristics (block 2); psychosocial factors (block 3); enrollment site and target disease markers (block 4); the eight SF-36 subscale scores (block 5); and self-reported disease severity (block 6).

## Results

### Patient and physician characteristics

Of the 1662 enrolled patients, there were 656 with CAD and/or CHF, 610 with COPD, and 396 with asthma. Enrollment was balanced among the Indianapolis (n = 838) and St. Louis (n = 824) sites. The study sample was 61.1% men, with a mean age of 63.1 years, and a racial distribution of 67.4% white, 28.4% black, and 4.2% non-white, non-black. On average, patients' socioeconomic status was low. The highest educational level obtained was grade school in 21%, some high school in 25.7%, high school graduate in 27.1%, some college in 18.4%, and college graduate in 7.8%. Half of the patients (50.5%) reported that they only had enough money to get by, and 25.9% reported that they did not have enough money to get by. These subjective income reports are consistent with more objective measures, including the fact that 43.6% of the patients reported having annual incomes below $15,000.

Nearly 84% of the patients reported a history of smoking cigarettes, with 63.1% having smoked for the equivalent of at least 20 pack years. As expected, smoking status was greatest among COPD patients, with over 80% having ≥ 20 pack years of smoking history, and less than 7% never having smoked cigarettes. There were few differences by enrollment site or target disease in terms of religiosity, social support, stress, sense of control, or patient satisfaction.

Forty-six primary care physicians participated in the study: 31 in Indianapolis and 15 in St. Louis. The different numbers of physicians enrolled by site reflects the greater proportion of time spent in patient care by physicians at the St. Louis site. This is further reflected in the average numbers of patients enrolled per PCP by site. In Indianapolis, the mean number of enrolled patients per physician was 27. In St. Louis, the mean number of enrolled patients per physician was 55.

### Physician estimates of disease severity

Table 1 summarizes mean PCP responses to the six baseline questions, both overall and by target disease and by enrollment site. Patient severity was rated as average, with the asthma patients considered just a little better than average. As expected, the predicted risks of hospitalization and death were also lowest among patients with asthma. Because patients with chronic heart or lung disease typically take some type of medication and because prescription of disease-specific medications was one mechanism for identifying potentially eligible patients through search of the electronic medical records, it is not surprising that medication rates were uniformly high. Testing and referral rates were lowest for the asthma patients, highest for the CAD/CHF patients, and higher among the patients from the Veterans Affairs Medical Center site.

**Table 1:** Baseline physician responses on six disease severity questions – overall and by target disease and enrollment site.

**Physician global and specific questions regarding severity of patient's disease**						
	**Severity^1^**	**Hospitalization^2^**	**Death^3^**	**Medications^4^**	**Tests^5^**	**Specialists^6^**
Overall	3.0	28%	15%	97%	84%	50%
Asthma	3.4	20%	9%	98%	65%	24%
CAD/CHF	3.0	31%	17%	98%	96%	78%
COPD	2.8	31%	17%	96%	83%	36%
Public Hospital	3.1	32%	16%	97%	73%	34%
Veterans Hospital	3.0	25%	14%	97%	94%	67%

^1^Compared to other patients that you see with <target disease>, how serious is this patient's condition (1 = much worse, ...5 = much better)?

^2^What is the chance (to the nearest 10%) that the patient will be hospitalized for <target disease> in the next 2 years?

^3^What is the chance (to the nearest 10%) that the patient will die, directly or indirectly due to <target disease>, in the next 2 years?

^4^Is this patient on medication(s) for his/her <target disease> (1 = yes, 0 = no)?

^5^As far as you know, has the patient had laboratory tests or procedures ordered because of his/her <target disease> (1 = yes, 0 = no)?

^6^As far as you know, has this patient seen a specialist for his/her <target disease> (1 = yes, 0 = no)?

### Physician's global estimate vs. specific disease severity variables

Table 2 depicts the association between the physician's global estimate of disease severity and the five more specific questions about disease severity. There was a classic bell-shaped distribution when patients (n = 1658) were categorized by their physician's response to the question: "Compared to other patients that you see with <target disease>, how serious is this patient's condition?" The PCP response to this global severity question was "much better" for 108 (6.4%) of the patients, "somewhat better" for 385 (22.7%), "about average" for 684 (40.3%), "somewhat worse" for 400 (23.6%), and "much worse" for 121 (7.1%).

**Table 2:** Association between physician's global estimate of disease severity and five physician-reported specific disease severity variables

	**Physician's Global Estimate of Disease Severity**				
**Physician-reported specific severity variables**	**Much Better**	**Some-what Better**	**About Average**	**Some-what Worse**	**Much Worse**
	(n = 108)	(n = 385)	(n = 644)	(n = 400)	(n = 121)
	----- percent -----				
Probability of hospitalization in the next 2 years *	8	14	24	45	72
Probability of death in the next two years *	6	7	12	21	48
Patient on disease medications*	83	96	98	100	99
Laboratory tests or procedures done *	54	70	88	96	99
Referred to specialist *	24	29	53	66	84

*p ≤ .001

Notably, there was a strong association between disease severity as assessed by the global question and by each of the five specific questions. Both the estimated probability of death and of hospitalization increased in a monotonic fashion as global estimate of disease severity worsened. Similarly, the proportion of patients who were on medications or who had received tests or referrals for their disease progressively increased as the global ("gestalt") estimate of disease severity worsened. Also, there was a good spread of responses for each of the five specific severity items from lowest to highest global severity category. The estimated 2-year probability of hospitalization ranged from 8% to 72% and of death from 6% to 48%. Likewise, the proportion of patients who had received tests ranged from 54% to 99%, and referrals from 24% to 88%. Only the proportion who were on medications demonstrated a more restricted range (83% to 100%). Together, these results suggest that the single-item global estimate of disease severity integrates more specific dimensions of disease severity and that its 5 response options reflect a broad range of severity.

### Association between patient self-rated health and physician assessments

Table 3 compares patient self-rated health with physician-rated disease severity, the probability of death, the probability of hospitalization, and whether the patient was on medications, had laboratory tests performed, or was referred to a specialist. In terms of self-rated health, 148 patients were in excellent or very good health (16.6%), 389 were in good health (23.5%), 640 were in fair health (39.9%), and 459 were in poor health (27.7%).

**Table 3:** Association between patient self-rated health and physician-reported disease severity variables

	**Patient's Self-Rated Health**			
**Physician-reported disease severity variables**	**Excellent or Very Good**	**Good**	**Fair**	**Poor**
	(n = 148)	(n = 389)	(n = 660)	(n = 459)
	------ percent ------			
Disease severity category*				
Better than average	38	39	31	24
About average	41	41	40	35
Worse than average	22	20	30	41
Probability of hospitalization in next 2 years *	24	24	27	35
Probability of death in next two years *	13	12	14	19
Patient on disease medications	97	97	98	97
Laboratory tests or procedures done	87	83	85	82
Referred to specialist	53	48	48	54

*p ≤ .001

There was a modest, direct association between patient self-related health and the physician's global ("gestalt") estimate of disease severity. That is, patients who self-rated their health as better were more likely to have physician estimates of less severe disease. There were also modest associations between patient self-rated health and the predicted probability of future hospitalization and mortality. Physician estimates of the 2-year probability of hospitalization ranged from 24% in the best category of self-rated health to 35% in the worst category. Similarly, the probability of mortality ranged from 12% to 19%. The biggest change in the probability of these two outcomes occurred between the fair and poor self-rated health categories. There was no relationship between patient self-rated health and three physician "action" items – medications, tests, and referrals.

### Patient variables associated with physician's global estimate of disease severity

Table 4 shows the patient variables that in multivariable models were independently associated with the physician's global estimate of disease severity. The beta coefficients were derived from the OLS models and the odds ratios from the multiple logistic regression models. The beta values reported in Table 4 are standardized (i.e., b coefficient is multiplied by the ratio of the standard deviation of the independent variable to the standard deviation of the dependent variable). Therefore, the magnitude of beta for a particular variable reflects the relative strength of its association with physician-estimated disease severity. The multinomial logistic regression model used patients with "average disease severity" as the reference group. For continuous variables, such as age in years or scale scores, the odds ratio for a particular category, as well as for between-category changes, appear small in magnitude because the OR is for each 1-unit change. Results were robust in that the same patient variables emerged as independent correlates in both linear and multinomial logistic regression models.

**Table 4:** Patient variables associated with physician's global estimate of disease severity †

	**Physician' Estimate of Disease Severity (Odds Ratio) ‡**				
**Patient Variable**	**Beta**	**Much Better**	**Somewhat Better**	**Somewhat Worse**	**Much Worse**
		(n = 108)	(n = 385)	(n = 400)	(n = 121)
**Increases physician's severity estimate**					
Age (older)	.173	0.950 ***	0.980 *	1.005	1.040*
Male	.166	0.597	0.720	1.666*	2.382*
Public Clinic	.128	0.996	1.500	2.468***	3.884***
Bodily Pain (less) ¶	.132	0.998	1.000	1.010**	1.028***
Long-term Smoker (≥ 20 pack-years)	.082	0.588***	0.724***	0.869	0.902
**Decreases physician's severity estimate**					
Physical Role Functioning (better) ¶	.153	1.004	1.004	0.994	0.968***
Disease Impact on Activities (less)	.137	1.051*	0.994	0.961**	0.927**
General Health Perception (better) ¶	.114	1.008	1.007	0.991	0.985
Retired	.112	2.355**	1.475*	0.907	0.797
Asthma	.109	1.853	1.217	0.599*	0.628

† Betas derived from ordinary least squares regression models, and odds ratios from multiple logistic regression models.

‡ Compared to reference group of 684 patients with "average disease severity". * = p < .05, ** = p < .01, *** = p < .001

¶ Scores on this scale range from 0 (worst health or function) to 100 (best health or function)

The overall variance in physician-estimated disease severity explained by the final OLS model was 16.4%. Examining the partial R-squared values, we found that demographics accounted for 3.7% of the explained variance (block 1); socioeconomic characteristics for 3.2% (block 2); psychosocial factors for 1.4% (block 3); site and target disease markers for 1.4% (block 4); generic HRQoL, i.e., the eight SF-36 subscales for 6.1% (block 5); and self-reported disease impact on activities for 1.0% (block 6).

Patient variables that were independently associated with an increase in the physician's global estimate of disease severity included older age, male gender, public hospital site, less severe bodily pain, and long-term smoking. Patient variables that were independently associated with a decrease in the physician's global estimates of disease severity included better physical role functioning or general health perceptions as well as those who were retired or who had asthma.

## Discussion

Our study of more than 1600 primary care patients with chronic heart or lung disease provided an excellent venue for examining a single-item physician-rated global estimate of disease severity. Several important findings emerged regarding this single item "gestalt" in which the physician is asked to compare the severity of a given patient's target condition to other patients seen with the same condition. First, the single item displayed a nearly perfect bell-shaped distribution of its 5 severity categories in all 3 medical conditions studied. This normal distribution provides face validity for the single item severity measure. Second, the global severity estimate was strongly associated with 5 more specific elements of disease severity – projected risks of hospitalization and mortality, and use of disease-specific medications, tests, and specialty referrals. Moreover, each of the specific measures showed substantial monotonic changes across the 5 categories of the global severity measure, confirming that the latter has good discrimination. This shows that the physicians were internally consistent in their different estimates of disease severity and acted in accordance with them. Third, both global and specific physician estimates of disease severity were only weakly associated with patient self-rated health, suggesting each may tap into somewhat different domains of illness burden. Finally, we identified patient variables that were associated with physician-rated disease severity.

Patient self-rated health has been shown in numerous studies to be a good single-item predictor of mortality, disability, and health care utilization [9-11]. Why a single estimate of health by patients is such a strong predictor of future health outcomes is not known but various hypotheses are discussed elsewhere [9,31]. In our study, the physician's estimate of disease severity was not strongly associated with patient self-rated health. Two previous studies comparing physician and patient global estimates had somewhat differing results. Maddox and Douglas compared self and physicians' assessment of general health status in a longitudinal study of 270 persons 60 years or older [12]. The two types of health ratings were positively correlated and, where incongruity did occur, individuals tended to rate their health more favorably than did physicians. Angel and Guarnaccia found dramatic discrepancies between physicians' and patients' assessment of patients' health, with patients' affective state, somatization, and language of interview further influencing this discordance [13].

One reason for the weak associations between physician and patient global ratings may be that physicians were asked to estimate the severity of a single target condition rather than the patient's overall health. Patients, on the other hand, were asked about their overall health and thus may have taken into account the sum effects of all their physical and psychological disorders. Along this line, patients were also asked to rate the severity of their target disorder in terms of the impact on five salient activities, and this measure was associated with physician-estimated disease severity (Table 4). Of note, recent research suggests generic measures that assess the impact and distress of health conditions from the patient's perspective may be useful across a variety of diseases [32,33]. A second reason may be that even when assessing the same condition, patients and physicians may focus on different factors or assign different weights to similar factors. For example, physicians may be more accurate in assessing objective measures of disease severity (including functional or physiological changes apparent only on physical examination or diagnostic testing) whereas patients may be more sensitive to symptoms and functional impairments that are not recognized or are under-appreciated by the physician. Regardless of the reasons for discordance, the fact that patient and physician ratings appear at least partly independent of one another means that both perspectives may be useful to researchers and clinicians.

The patient factors found to be associated with physician-estimated disease severity were consistent in both linear and logistic regression models. Nonetheless, these results should be considered the most exploratory of our findings. Some of the patient variables have face validity in their association with physician-estimated disease severity, such as physical role functioning, patient self-rated disease impact on activities, general health perceptions, and long-term smoking. Also, asthma is a more episodic condition than either CAD/CHF or COPD, being manifested in many patients with quiescent periods of varying duration rather than chronic daily symptoms or progressive deterioration. Demographic variables such as age and gender might be associated with prognostic factors physicians consider but were not measured in this study. At the same time, it is important that age or gender bias does not lead physicians to overestimate disease severity in older patients or underestimate it in women. Although greater severity estimates for patients at the public hospital site could theoretically be due to physician attitudes or unmeasured patient factors, this is confounded by differences in geographic location as well as substantially different clinical workloads of physicians at the two sites. The fact that retired subjects had better physician-rated health could reflect, in part, selection bias.

Compared to their skills in diagnosis and therapy, physicians feel less comfortable with their prognostic abilities [34,35]. The modest research conducted in this area has been principally in seriously ill hospitalized or terminally ill cancer patients [14-19]. One study found that physician estimates had an independent effect beyond models incorporating other risk factors in predicting survival in patients with coronary disease [36]. Another study revealed that in patients presenting to an emergency department with chest pain, the physician's global estimate of the likelihood of myocardial infarction was the single strongest predictor of the patient actually having an infarction [37]. Other investigators found that in predicting return to work in patients with coronary disease, both physicians' and patients' estimates had independent prognostic value [38]. However, physicians relied predominantly on medical variables (cardiac status and comorbidity) whereas patients' estimates were based on overall health status as well as job-related variables. Finally, the physician's estimate of whether laboratory tests will be abnormal has independent predictive value beyond other clinical data [39].

Our study has several limitations. First, as already mentioned, physicians were asked to rate severity of the patients' target disease whereas patients provided global rating of their overall health. Second, all variables were physician and patient-reported measures. Though physicians likely incorporated knowledge of physiological or anatomic tests in their severity ratings, and patients completed a rich inventory of generic and disease-specific HRQoL measures, certain objective data (e.g., coronary anatomy, systolic function, spirometry) might provide independent information on disease severity that could prove useful in future validation studies. Third, our analyses relied on data gathered at one time point (i.e., upon study enrollment), meaning that all associations are cross-sectional rather than longitudinal. Prospective studies would be important to examine the predictive validity of physician-rated disease severity for outcomes such as hospitalization, disease progression, health care utilization, and mortality. Fourth, since each patient's PCP answered both the global and specific severity questions, responses to these six items are not independent, possibly inflating the associations in Table 2. Fifth, the specific severity items themselves are somewhat interdependent in that patients with a higher projected mortality risk may also be more likely to be hospitalized and receive medications, diagnostic tests, and subspecialty referrals. Nonetheless, the association of each of these specific factors with the physician's global severity estimate does provide evidence for convergent validity.

Future research should examine the predictive validity of physician-rated disease severity, including how well it compares with other comorbidity measures [40] as well as patient self-rated health [9-11]. The question is not only whether certain measures have superior prognostic value but also whether they contribute independent information such that, when combined, their predictive value is additive. Further, the factors that influence physician estimates of disease severity should be parceled out, examining not only variables we found as correlates but also factors not examined in our study. Like patient self-rated health, physician-estimated disease severity may prove to be a simply assessed yet powerful predictor of future outcomes. Health status assessment is neither an exclusively patient-centered nor physician-driven process but rather an integration of important input from both parties.

## Conclusion

Physicians' global estimates of patients' disease severity are strongly associated with their estimates of more specific aspects of disease severity such as diagnostic and treatment actions and projected risk of hospitalization and mortality. However, physicians' and patients' global estimates are only weakly correlated. Despite important limitations of our study, these preliminary findings suggest physicians and patients may weight different aspects of disease severity and incorporating both perspectives in clinical decision making and outcomes research may be important.

## Competing interests

The author(s) declare that they have no competing interests.

## Authors' contributions

KK participated in acquisition and interpretation of data, and drafting of the manuscript. KWW was involved in conceptualizing the study design and acquisition of data. WMT and ANB participated in acquisition and interpretation of data. FDW conceptualized the rationale and design of the study and performed the statistical analysis. All authors read and approved the final manuscript.
